# Cyanovirin-N Binds Viral Envelope Proteins at the Low-Affinity Carbohydrate Binding Site without Direct Virus Neutralization Ability

**DOI:** 10.3390/molecules26123621

**Published:** 2021-06-13

**Authors:** Irene Maier, Robert H. Schiestl, Georg Kontaxis

**Affiliations:** 1 Department of Environmental Health Sciences, Fielding School of Public Health, University of California, Los Angeles, CA 90095, USA; RSchiestl@mednet.ucla.edu; 2 Max Perutz Laboratories, Department of Structural and Computational Biology, University of Vienna, A-1030 Vienna, Austria; georg.kontaxis@univie.ac.at

**Keywords:** cyanovirin-N, human immunodeficiency virus, hemagglutinin, ebola virus, surface plasmon resonance, high-mannose glycan, glycoprotein

## Abstract

Glycan-targeting antibodies and pseudo-antibodies have been extensively studied for their stoichiometry, avidity, and their interactions with the rapidly modifying glycan shield of influenza A. Broadly neutralizing antiviral agents bind in the same order when they neutralize enveloped viruses regardless of the location of epitopes to the host receptor binding site. Herein, we investigated the binding of cyanovirin-N (CV–N) to surface-expressed glycoproteins such as those of human immunodeficiency virus (HIV) gp120, hemagglutinin (HA), and Ebola (GP)1,2 and compared their binding affinities with the binding response to the trimer-folded gp140 using surface plasmon resonance (SPR). Binding-site knockout variants of an engineered dimeric CV–N molecule (CVN2) revealed a binding affinity that correlated with the number of (high-) affinity binding sites. Binding curves were specific for the interaction with N-linked glycans upon binding with two low-affinity carbohydrate binding sites. This biologically active assembly of a domain-swapped CVN2, or monomeric CV–N, bound to HA with a maximum K_D_ of 2.7 nM. All three envelope spike proteins were recognized at a nanomolar K_D_, whereas binding to HIV neutralizing 2G12 by targeting HA and Ebola GP1,2 was measured in the µM range and specific for the bivalent binding scheme in SPR. In conclusion, invariant structural protein patterns provide a substrate for affinity maturation in the membrane-anchored HA regions, as well as the glycan shield on the membrane-distal HA top part. They can also induce high-affinity binding in antiviral CV–N to HA at two sites, and CVN2 binding is achieved at low-affinity binding sites.

## 1. Introduction

The ability of lectins, such as cyanovirin-N (CV–N), and antiviral carbohydrate-binding agents to bind highly conserved epitopes on the influenza hemagglutinin (HA) glycoprotein is of key importance to the rational design of vaccines for prophylactic and therapeutic use [1,2,3]. To elucidate the stoichiometry of bound molecules and their interactions with the rapidly modifying glycan shields, glycan-targeting antibodies have been studied for binding and broad neutralization efficacy to influenza A H3N2 [4] and H1N1 subtypes [4,5,6]. Moreover, it has been observed that oligosaccharide modification of tetherin N-linked glycosylation is necessary and sufficient for tetherin-mediated restriction of HIV-1 release [7], but it remains controversial in several other studies. The importance of using defined glycosylation patterns has been demonstrated in vitro, which in turn depends on the conformation of the surface-expressed HA membrane-fusion active part in the latter [8]. Among others, the surface-expressed envelope protein HA and the neuraminidase on influenza A virus are known for tetherin antagonism in a strain-specific manner [9], as they facilitate recognition of host receptor binding sites [10,11]. To study the glycosylation pattern, a binding-site specific tool such as the domain-swapped anti-HIV 2G12 antibody (Ab) attachment had to be used. The domain-swapped anti-HIV neutralizing Ab (NAb) complexed with Man(9)GlcNAc(2), was co-crystallized with Man(4), Man(5), Man(7), and Man(8) moieties [12], and has been found specific for Manα1-2Man binding [13], but capable of binding both the D1 and D3 arms of the Man(9)GlcNAc(2) moiety. The specificity of this Ab in dissecting carbohydrate binding provides the necessary flexibility to make the required multivalent interactions between the Ab and the gp120 oligomannose cluster [12,13]. Prior research investigated epitopes and structures of HA membrane-distal and stem regions that were interacting with influenza NAbs and monoclonal antibody (mAb) fragments directed to HA regardless of their location of epitopes in relation to the receptor binding site [10,14,15].

CV–N is a cyanobacterial lectin, which acts like a pseudo-antibody, thus providing a scaffold for determining how the lectin binds by using variants of different computationally designed stabilities and how certain functional groups affect binding to the viral envelope spikes of, for example, HIV-1 gp120, HA and Ebola virus glycoprotein (GP) [9,16,17]. To mimic molecular interactions with conserved epitopes on viral GPs, we chemically synthesized di- and tri-mannose moieties via the azidoglycosylation of glycosyl β-peracetates to 1,2-trans glycosyl azide transformation as developed by Salunke et al. [18]. Mannosylated influenza glycoprotein peptides were prepared, mimicking the naturally found N-acetyl glucosamine and high-mannose oligosaccharides on the surface of viruses that cause life-threatening viral infections [19]. We used triazole bioisosteres as linkages to form the mannosylated residue of HA peptide sequence, which facilitated site-specific interactions with CV–N derivatives around the second N-linked glycosylation site on the HA head domain (HA top comprising 4 N-linked glycans N54, N97, N181, N301) [20,21]. In the present study, we tested the binding affinity of wild-type CV–N (WT CV–N) and engineered a domain-swapped dimer against viral spike proteins which were either expressed in mammalian or insect cells using surface plasmon resonance (SPR) spectroscopy.

## 2. Results

**Figure 1:**
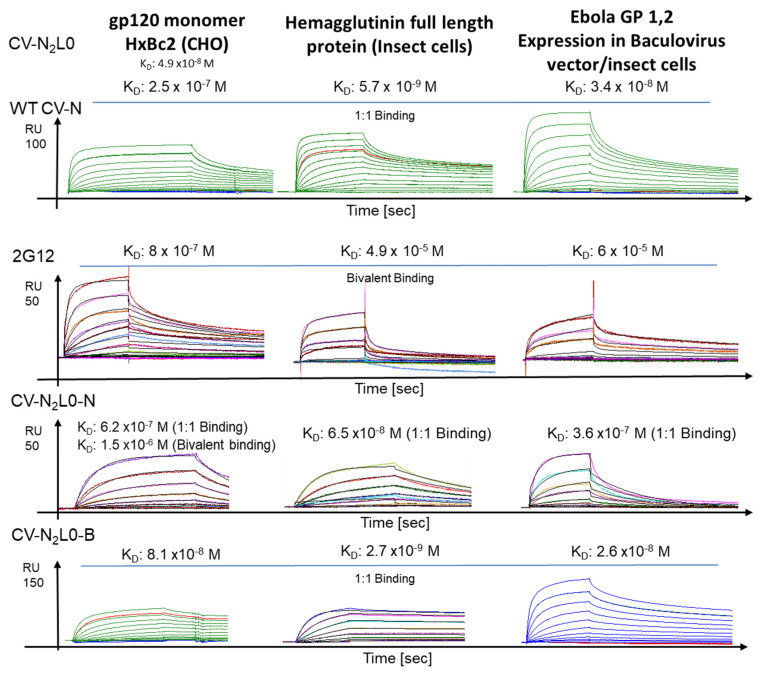
CV–N, 2G12, and CVN2L0-N and -B binding to HIV gp120, HA and Ebola GP1,2. SPR binding analysis of CV–N monomer, 2G12 binding, and tandem-linked domain swapped dimer binding with 2L, and dimer with 2H + 1L. Equilibrium dissociation constant (K_D_) for interactions of CVN2L0 with HIV-1 gp120 was 4.9 × 10^−8^ M (this study) and with HA, 2.55 × 10^−7^ M [19]. Kinetic data set (5120, 2560, 1280, 640, 320, 160, 80, 40, 20, 10, 5, 2.5, 0 nM) showed real-time binding to gp120, influenza HA A/New-York/55/04 (H3N2), and Ebola GP1,2 at nanomolar K_D_ using the 1:1 binding model for CV–N and mutants, and bivalent 2G12 binding to gp120. K_D_ for specific 2G12 binding to HA and Ebola GP1,2 was calculated by simulating bivalent binding in µ-molar. All data were achieved on a CM5 sensor chip using BIAcore T100 at 25 °C with a flow run of 30 µL/min.

The low binding affinities of anti-HIV NAb 2G12 to the influenza HA full-length protein and Ebola GP1,2 were evaluated in the µ-molar range for avidity as compared to the binding of HIV-1 gp120, and CV–N binding to viral GPs. Furthermore, an engineered dimeric CVN2L0 molecule bound gp120 with an equilibrium dissociation constant (K_D_) of 49 nM. WT CV–N monomer binding was at K_D_ = 250 nM to gp120, 5.7 nM to HA and 34 nM to Ebola GP1,2. Next, we used several variants of a combination of one to seven knockout mutations to diminish and measure altered affinity to carbohydrate binding domains A or B, respectively. WT CV–N and CNV2L0-N had an even number of binding sites, namely, two. Whereas a single high-affinity binding site (H) and low-affinity binding site (L) were associated with WT CV–N, CVN2L0-N represented a linker-free domain-swapped dimer with two binding sites of equally binding low-affinity sites (Figure 1 and Figure S5). Using the SPR bivalent binding model for these K_D_ calculations, the binding site knockout variant CVN2L0-N recognized gp120 at K_D_ = 1.5 µM, and the binding of 2G12 to gp120 was at K_D_ = 800 nM (Figure 1). Therefore, the Ab was comparably higher in binding affinity than the interaction between gp120 and dimeric pseudo-Ab CVN2L0-N, assuming a low-affinity to N-glycans for CVN2L0-N. Higher avidity with the number of functional H in CVN2L0 was achieved in conjunction with another L in CVN2L0-B (Table 1 and Figures S1, S2, S4 and S5). Binding site knockout variants -B, -N, and -E were functionalized with either 3, 2, or 1 binding site(s). Dimeric CVN2L0-B and monomeric CVN-E bound with 2H and 1H, respectively. CVN2L0-B had additionally one L, which did not add much to avidity to Ebola GP1,2, but to HA, if compared to CVN-E (1H), or WT CV–N (1H + 1L). CVN-E binding to HA was 100-fold weaker than CVN2L0-B binding to HA and 10-fold weaker to HIV-1 spike proteins than CVN2L0-B. A single H was dominant for the interaction of CV–N to envelope proteins from all three viruses in the nanomolar range (Table 1). This effect applied to the interactions of HA with the dimeric variant CVN2L0-V2, which had an abolished disulfide bond that was exchanged for ion-pairing amino acids C58E and C73R [19] (Figure S2). To investigate low-affinity binding to HA and Ebola GP1,2, the 1:1 binding model was evaluated among sensorgrams, which did not fit the CVN2L0-N dissociation rate (Figure 1, Figures S2 and S4). CVN2L0 variants showed the highest affinity for HA (Figure S2) over Ebola GP1,2, gp120 and the gp140 trimer (Table 1, Figures S1 and S4). CV–N and CVN2 binding affinity was achieved in the nanomolar range to A/Wisconsin/67/05 and A/New-York/55/04 (both H3N2) on SPR (Figure S2) and compared anti-HA HC19 and CV–N binding to HA H3 top (Figure S3) in SPR with enzyme-linked immunoassays (K_D_ = 1.25 × 10^−8^ M for HC19 vs. 5.54 × 10^−7^ M for CV–N, Figure S5). In addition, HC19 anti-influenza Fab bound to HA top with K_D_ = k_d_/k_a_ = 64 nM in the SPR experiment (association rate constant, k_a_ [M^−1^ s^−1^] = 3.6 × 10^5^; dissociation rate constant, k_d_ [s^−1^] = 0.02312) and to escape mutants in the µ-molar range [20].

## 3. Discussion

### 3.1. CV–N and 2G12 Binding Affinities for HIV gp120, HIV gp140, HA, and Ebola GP1,2

We examined the binding affinities and avidity of anti-HIV neutralizing 2G12 to an influenza HA full-length protein and Ebola GP1,2 in the mid µM range (Figure 1). The binding of 2G12 to glycan moieties was previously described to be higher to monosaccharide D-fructose than D-mannose [22], and its binding to gp120 can be inhibited by lectin actinohivin [23]. In contrast, CV–N binding has been shown to dimannose and trimannose-conjugates [24], but the broad neutralization ability of antiviral agents alike CV-N resulted from interactions with high-mannose containing oligosaccharides, found on, for example, HA [9,17]. In this study, we compared CV–N binding between the gp120 monomer and the trimer-folded gp140, HA, and Ebola GP1,2 envelope spikes. There were no differences found in the specificity of CV–N binding towards high-mannose, hybrid-, or complex-type glycans according to the expression system used to produce recombinant gene products for these binding studies (Figure 1). An interaction with a variety of sugars was reported concerning 2G12, and we successfully showed cross-reactivity of domain-swapped 2G12 against HA and Ebola GP1,2 to reveal specific binding and broad affinity determination. CV–N and CVN2L0′s HIV neutralization ability, however, was mainly attributed to the recognition of Manα1-2Man residues on high-mannose oligosaccharides on the enveloped virus [25,26], and restored by dimerization of binding-site knockout constructs [27]. A stable domain-swapped molecule was achieved that allowed refinement of crystallographic studies and the high-resolution display of carbohydrate binding sites [26]. Other cyanobacterial lectins, such as scytovirin (SVN), microvirin (MVN), *Microcystis viridis* lectin (MVL), and *Oscillatoria agardhii* agglutinin (OAA), as well as cyanobacterial extracts, polysaccharides, peptides, and other metabolites also have potential as antiviral drugs with various specificity to high-mannose oligosaccharides [2].

Similar to HIV gp120/gp41, Ebola GP1 affects attachment to host cells, whereas GP2 mediates fusion of viral and host membranes forming the trimer-fold of a spike protein on enveloped viruses, which was found to be a target for CV–N antiviral activity to abolish ex vivo and in vivo viral cytopathic effects in mice [28]. Ab fragment binding further explored Fab binding and provided evidence for binding-active structures to Ebola virus GP from a human survivor of Ebola virus infection [29]. Ebola virus, however, is thought to enter host cells by receptor-mediated endocytosis through clathrin-coated pits and caveolae, followed by actin- and microtubule-dependent transport to the endosome [30,31]. The expression in baculovirus vector-infected insect cells showed evidence of a surface envelope Zaire GP to be synthesized and glycosylated and that this protein similarly bound CVN2 to HIV-1 gp120 alone or to gp140 (Table 1, Figures S1 and S4). Both signal motifs and posttranslational modifications such as glycosylation determine whether a protein in the late endosome will be incorporated into vesicles destined for the trans-Golgi or lysosome [32]. Ebola GP was found to have accumulated in the endoplasmic reticulum [33]. The main viral determinant of Ebola virus pathogenicity, inducing cytotoxic effects in human endothelial cells, is still uncertain but usually associated with the intracellular synthesis or transport of the gene product of the Ebola virus surface virion GP to the cell surface.

### 3.2. Avidity Correlated with the Number of CV–N Carbohydrate Binding Sites to Recognize Spike Proteins

**Figure 2:**
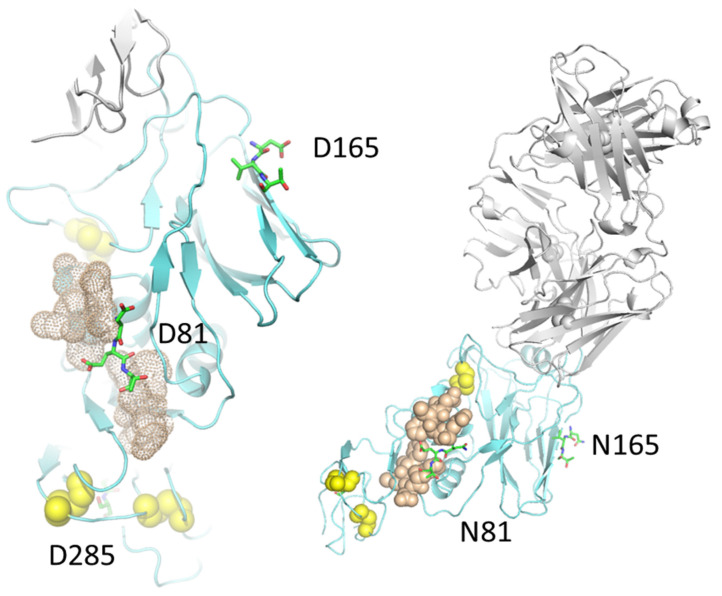
HC19 Fab bound to HA top showing HA and in silico Asn(N) mutated glycosylation sites on HA top domain, namely N81, N165, and N285 as sticks (structure on the right). A 12-mer short peptide (shown as brown dots), which was chemically linked to azido-dimannose, has been shown to bind CVN2L0-V2 [19], and comprises residues C76 to Q80 and E82 to F87. Disulfide bond-forming Cys residues in HA are shown as yellow spheres. Left structure: Part of X-ray structure PDB ID: 2VIR showing chain C HA of influenza A virus (X-31) in cyan, with a molecular distance of 30.8 Å between D81 and D165, and a distance of 34 Å between D81 and D285.

Several studies correlated CVN2 binding affinity with the number of functional binding sites: Two high-affinity carbohydrate binding sites on domain B, located distal from the N and C termini, and two low-affinity carbohydrate sites on domain A [34,35] and disulfide bonds [19]. The crystal structure of CVN2 shows a flexible linker, or hinge, and two sequence-based domains that form a dimer by intermolecular domain-swapping. Among dimannose interacting residues, E41 is involved in glycan binding, as an intramolecular domain-linker [36], and as a residue located in the high-affinity pocket that was mutated for binding site knockout variants. Based on computational protein design investigations, we made new glycan-interacting homodimeric CVN2L0 scaffolds to probe the binding capacities at mannose-recognizing low-affinity carbohydrate binding sites. As the number of disulfide bridges near the glycan-binding pocket decreased from 4 to 2 by symmetrical substitution of Cys and insertion of polar residue pairs Glu–Arg, the binding affinity to HA protein decreased [19]. N-terminal Cys (C)–Asp (D) and another 7 residues spaced N-terminal Cys from C-terminal Phe (F) around the protein derivative, forming the glycosylation site (Asn/Gly–Glu–Thr) on mannosylated peptides (Figure 2). A third CV–N complementary pseudo-domain was formed in vitro to recognize HA by this lectin involving C76, a cysteine of a disulfide bond in the HA target that is a possible site for polar interactions with the dimannose moieties or a substitution of C58–C73 in CV–N. First, we showed preferences for CV–N binding to dimannose units using SPR and isothermal titration calorimetry (K_D1_ = 306 nM for CVN2 high-affinity carbohydrate binding site; K_D2_ = 4 µM for low-affinity carbohydrate binding site) [19]. We varied the number of mannose–mannose linkages in the target, deciphering interactions with tryptophan in the high-affinity glycan pocket using saturation transfer difference–nuclear magnetic resonance (STD-NMR). Multivalent interactions with dimeric CVN2L0-B were attributed to either the low-affinity carbohydrate binding site comparing its role in WT CV–N and unmutated CVN2L0 or a conformational change to stabilize the disrupted high-affinity binding pocket in variant 2 (minus a disulfide bond; K_D_ = 49 nM to HA, Figure S2). The targeted saccharides may have acted as multiple ligands when exposed on spike proteins. CVN2L0-P with 1H in the dimer did not show sufficient binding, nor was one carbohydrate binding site sufficient for achieving neutralization [26,27]. Secondly, binding studies between mannose-linked HA peptide and domain-swapped CVN2 based on NMR indicated that this protein dimer cross-linked on two potential low-affinity carbohydrate binding sites. A region of complementary binding-site residues around a naturally found glycan on influenza HA (H3N2) top were used and intrinsically integrated into the development of medium-throughput screening methods for glycans that target broadly neutralizing antiviral agents that recognize viral escape mutants. Glycosylation of the HA-neuraminidase (HN) antigenic site demonstrated diminished viral fusion and a consequent deficiency in syncytium formation in the infected cells, where the virus can escape the neutralizing effect of monoclonal antibodies by adding an N-glycan at the D287N-mutated HN site in the baby hamster kidney cell transient expression system [37]. So far, HA has been found to trigger B-cell antigen receptor (BCR)-associated tyrosine kinase signaling by means of germline transmembrane immunoglobulin (Ig)-M but was not bound as soluble IgG (conjugate). Selected Ig genes recognize specific and invariant structural protein patterns that provide a substrate for affinity maturation in the HA transmembrane stem region [38]. In comparison, the higher affinity of Fc mutants to the receptor for eliciting effector functions in vivo, revealed the composition of hybrid-type N-linked glycans attached to Fc mutants to be mechanistically determined [39].

In conclusion, HA binding was examined for an intact fold of 1H in CV–N, and 2H in dimeric CVN2 in conjunction with another L at K_D_ = 5.7 nM and K_D_ = 2.7 nM, respectively. Ebola GP1,2 bound CV–N predominantly with high-affinity carbohydrate binding sites in the range of 26–72 nM in either CVN2L0, or CV–N. The CVN2L0-B variant with two unmutated high-affinity carbohydrate binding sites showed the highest affinity to all tested viral GPs in this study. A variety of polar interactions and interaction sites were localized in CV–N carbohydrate binding pockets involving low-affinity contributing sites in domain A and are currently accomplished by molecular dynamics simulations, but further investigation is needed to understand the impact of immune-suppressive N-linked glycosylation around the host receptor binding site.

## 4. Materials and Methods

### 4.1. Protein Expression and Purification

The gene for WT CV–N was constructed using a recursive PCR method with 40-mer synthesized oligos [40] and then subcloned into the NdeI and BamHI sites of pET11a as described previously [26]. The protein contained an N-terminal 6-histidine purification tag followed by a Factor Xa protease cleavage site. Binding-site knockout mutant constructs (B, N, P; Figures S1 and S5) were generated in the background of a CVN2L0 template gene containing two distinct DNA sequences for each CV–N repeat [26] or monomeric CV–N in case of CVN-E (Figure S1). Mutations were made using the QuikChange Multi Site-Directed Mutagenesis Kit (Stratagene, San Diego, CA, USA). See also Supplementary Material.

The expression of WT CV–N and dimeric molecules CVN2L0, binding-site mutants, and disulfide bridge variants, was induced with IPTG in BL21 (DE3) *E. coli* cells in LB including ampicillin. The harvested cells were lysed using an EmulsiFlex-C5 (Avestin, Inc., Ottawa, ON, Canada), and the insoluble fraction was resuspended in a buffer containing 6 M GuHCl and 10 mM imidazole and centrifuged to remove debris. The solubilized CV–N was then purified under denaturing conditions using a Ni-NTA gravity column (Qiagen, Hilden, Germany) and refolded by dialyzing the Ni-NTA eluate against a native buffer overnight at room temperature. Following refolding, the proteins were additionally purified on a Superdex-75 column and eluted in 25 mM sodium phosphate pH 7.4, 150 mM NaCl, concentrated, and stored as eluted at 4 °C.

Ebola virus expresses 2 types of GP molecule from a single gene. The primary gene product is a C-terminally truncated 364-residue surface glycoprotein (sGP) that is released from infected cells. The full-length 676-residue form of the GP, which is incorporated into the virion envelope, is expressed as a result of transcriptional editing, which results in the addition of an extra non-templated adenosine within a run of seven adenosines near the middle of the coding region [41]. The Ebola surface envelope protein GP1,2 consists of two subunits GP1 and the membrane anchored GP2, which are covalently linked by disulfide bonds as GP1,2 and form a spike on the virion surface. GP is post-translationally cleaved by furin. Synthesis of the virion GP of Ebola virus Zaire [28,42] induced cytotoxic effects in human endothelial cells and was therefore expressed in insect cells with a baculovirus carrying the recombinant gene [29] assembled as described in [40]. The construct used in this study kept the mucin-like domain intact. Neither the signal peptide (residues 1–33), nor the transmembrane domain was expressed.

Other viral glycoproteins were expressed in either mammalian cells (2G12, HC19, HA top domain, HIV gp120) or baculovirus-infected insect cells (gp140 trimer Clade A 92 UG 037.8). HA full length proteins (A/New-York/55/04, A/Wisconsin/67/05) were purchased from abcam (Cambridge, UK). The expression vector pFastBac-1 was used for gp140 proteins and Bac-to-Bac system (Invitrogen, Carlsbad, CA, USA) as described before [43]. *Trichoplusia ni* (Hi-5) cells (2 × 10^6^ cells per mL) were infected at optimal multiplicity. We used PyMol (The PyMol Molecular Graphics System) for molecular representation of the crystal structures of influenza HA glycoproteins and NAb.

### 4.2. Expression of 2G12, HC19 Fab and HA H3 Top

Gene constructs were subcloned into the mammalian expression vector pTT5 (NRC Biotechnology Research Institute, Montréal, QC, Canada) for expression using the polyethylenimine-mediated transient transfection for suspension cultured HEK293-6E cells. The 2G12 [44] (and HC19 Fab [20]) heavy- and light-chain expression vectors were co-transfected at a 1:1 ratio using 25-kDa linear polyethylenimine (Polysciences, Warrington, PA, USA) [44]. Cell culture supernatants were collected at 6 days post transfection, passed over protein A resin (Thermo Fisher Scientific, Waltham, MA, USA), immediately neutralized, and then subjected to size exclusion chromatography in 20 mM Tris (pH 8.0)-150 mM NaCl using a Superdex 200 16/60 or 10/30 column (GE Healthcare, Chicago, IL, USA). Sequences encoding the HA H3 top domain in the pTT5 vector were expressed in mammalian HEK293T cells and purified via His-tag on Ni-NTA columns.

### 4.3. HIV-1 Envelope Spike Proteins

The Gp120 monomer, strain HxBc2, was either expressed in mammalian CHO or HEK293 cells, whereas the gp140 trimer was expressed in Hi5 insect cells according to protocols used by the Caltech Protein Expression Center.

### 4.4. SPR Binding Studies

A BIAcore T100 biosensor system (GE Healthcare, Chicago, IL, USA) was used to evaluate the binding affinities and avidity of the domain-swapped dimer CVN2L0 to gp120. Moreover, WT CV–N (as well as CVN2L0-N and -B binding-site mutants), and 2G12 were measured for binding to gp120, gp140, HA, and Ebola GP1,2. In this system, a protein was coupled to a gold-dextran layer, and association and dissociation phases for binding to an injected protein were measured in real time in resonance units (RU). The WT CV–N monomer reached at least two times the response (~100 RU) of low-affinity CVN2L0-N and 2G12 (∼50 RU of each) that were captured onto ∼2500 RU of the viral glycoprotein, which was immobilized by primary amine coupling to a CM5 sensor chip as described in the BIAcore manual. A concentration series of monomeric CV–N, binding-site mutants, disulfide bridge variants, and 2G12 were injected at 30 μL/min over the flow cells. After the dissociation phase, the surface was regenerated by the injection of pH 1.5 glycine buffers. HC19 Fab was captured to the Series S sensor chip Protein A (GE Healthcare, Chicago, IL, USA) prior to subsequent HA H3 top injection; experiments were repeated after two regeneration steps by injecting a pH 1.5 glycine buffer.

The disulfide bond variant CVN2L0-V2 was tested for binding to HA, and studies were performed on Reichert’s SR7500DC (Reichert, Buffalo, NY, USA), a two-channel SPR instrument. CMD500D SPR sensorchips were purchased from Xantec biosensors (Düsseldorf, Germany). Kinetic studies were performed using various analyte concentrations in the range of 10^−5^–10^−8^ M, with a regeneration step after each injection, and blank measurements after different analytes. The running buffer (HBS-EP (+)) contained 10 mM HEPES, 150 mM NaCl, 3 mM EDTA and 0.05% Tween at a pH of 7.4 (see Supplementary Materials).

### 4.5. Enzyme-Linked Immunosorbent Assay

Nunc Maxisorp microtitration plates were coated with HA H3 100–0.01 μg/mL, blocked with 1% bovine serum albumin, washed 3 times with PBS-Tween, and subsequently incubated with a SUMO-CVN2L0 fusion protein. Anti-SUMO primary antibody (1:1000) and secondary anti-Chicken IgY antibody conjugated with horseradish peroxidase (HRP) (1:5000) were used for the read out. Washing (3–5 times) was applied after each incubation step. An anti-HA HC19 wt antibody was used as the primary antibody (1:2000) to detect HA H3 directly, after which anti-human IgG-HRP was applied at a dilution of 1:30,000 and TMB (Sigma-Aldrich, St. Louis, MO, USA) for read-out.

## Figures and Tables

**Table 1:** Comparison of CV–N affinities to enveloped virus spike glycoproteins.

		HIV-1 K_D_ (M)		Influenza K_D_ (M)	Ebola K_D_ (M)
		Monomer	Trimer-Folded		
WT CV–N	1H + 1L	2.6 × 10^−7^	1.2 × 10^−7^	5.7 × 10^−9^	3.4 × 10^−8^
CVN_2_L0-B	2H + 1L	4.3 × 10^−8^	7.2 × 10^−8^	2.7 × 10^−9^	2.6 × 10^−8^
CVN_2_L0-N	2L	1.8 × 10^−7^	3.5 × 10^−7^	6.5 × 10^−8^	3.6 × 10^−7^
CVN E	1H	4.5 × 10^−7^	7.2 × 10^−7^	2.0 × 10^−7^	7.2 × 10^−8^

WT CV–N and CNV2L0-N had an even number of binding sites (2). Binding-site variants based on CVN2L0 -B, -N, and -E were functionalized with 3, 2, and 1 binding sites, respectively. WT CV–N and CVN-E based on WT CV–N monomer had the high-affinity binding site (H) and the low-affinity binding site (L) or H.

## Data Availability

Not applicable.

## Supplementary Materials

The following are available online: Figure S1: Surface Plasmon Resonance. Binding assays showing binding of CV–N and variants to gp120 and gp140, Figure S2: Surface Plasmon Resonance. Binding of CV–N and variants to HA, Figure S3: Surface Plasmon Resonance. Binding of HC19 Fab and CV–N to HA top, Figure S4: Surface Plasmon Resonance. Binding of CV–N variants to Ebola GP1,2, Figure S5: Immunoassay for the binding of CV–N and variants to HA top and HA full length protein.

## Sample Availability

Samples of the compounds are available from the authors.
