# Supplementary material for: Cyanovirin-N Binds Viral Envelope Proteins at the Low-Affinity Carbohydrate Binding Site without Direct Virus Neutralization Ability
Source: Molecules. 2021 Jun 13;26(12):3621. doi: 10.3390/molecules26123621 (PMC8231982; doi:10.3390/molecules26123621)
Supplement: Supplementary file 1 [file molecules-26-03621-s001.zip › molecules-1219230 SF.pdf]

molecules

MDPI

Supplementary Material

Cyanovirin-N Binds Viral Envelope Proteins at the Low-Affinity Carbohydrate Binding Site without Direct Virus Neutralization Ability

Irene Maier [1,*], Robert H. Schiestl [1] and Georg Kontaxis [2]

[1] Department of Environmental Health Sciences, Fielding School of Public Health, University of California, Los Angeles, CA 90095, USA; RSchiestl@mednet.ucla.edu
[2] Max Perutz Laboratories, Department of Structural and Computational Biology, University of Vienna, A-1030 Vienna, Austria; georg.kontaxis@univie.ac.at
* Correspondence: irene.maier@meduniwien.ac.at; Tel.: +1-310-267-2087; Fax: +1-310-267-2578

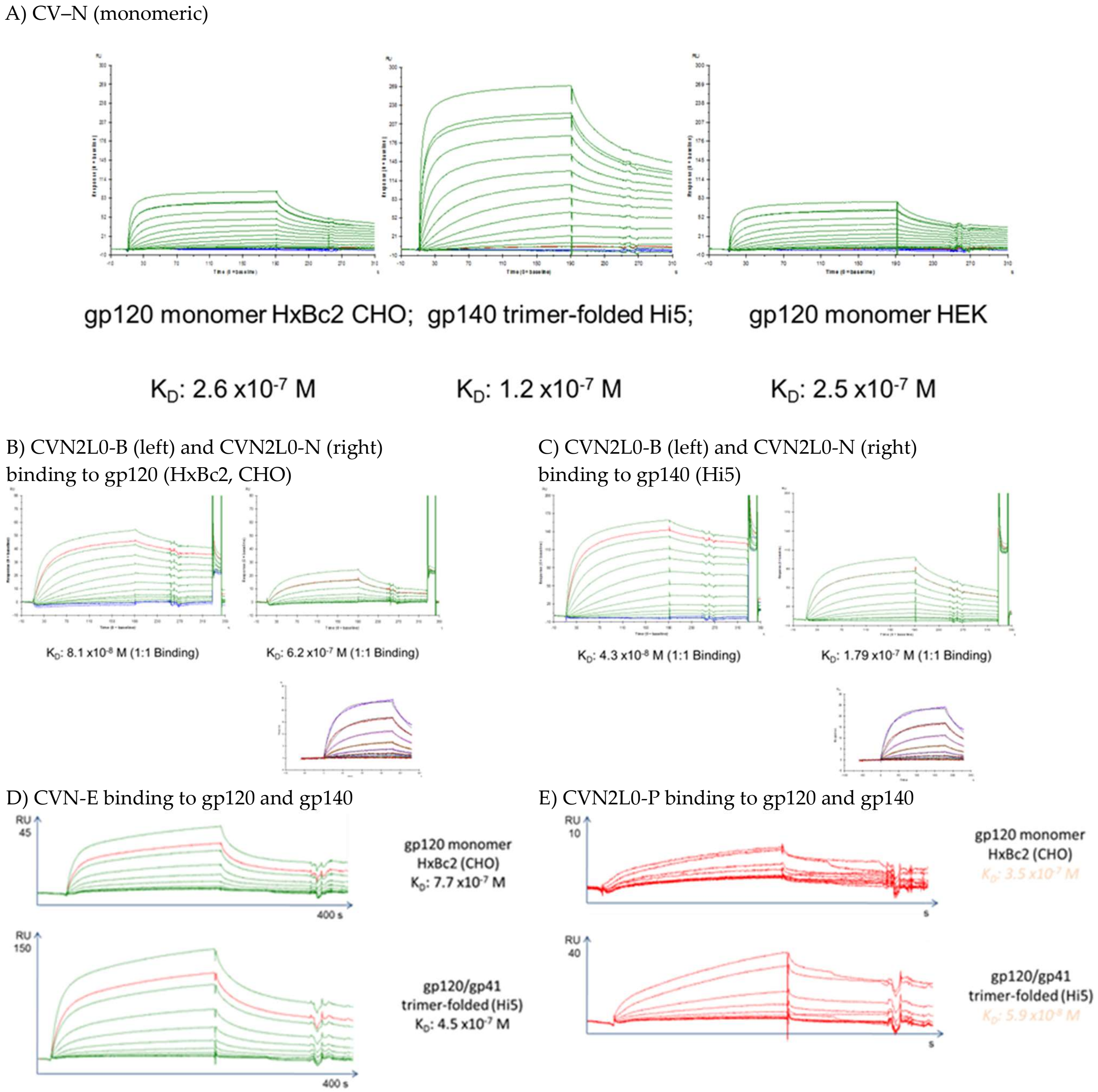

Figure S1. Surface Plasmon Resonance (**A**) CV–N binding curves are shown for binding to HIV-1 envelope S glycoproteins gp120 expressed in mammalian CHO or HEK293 cells, and the gp140 trimer-folded spike. (**B**) Sensorgrams from analyses in which binding site knockout mutants CVN2L0-B (2 high-affinity binding sites-H and 1 low-affinity binding site-L) and CVN2L0-N (2 L) were injected over immobilized gp120. Concentrations of analytes ranged from 5120 nM (2560, 1280, 640, 320, 160, 80, 40, 20, 10, 5) to 2.5 nM. (**C**) Sensorgrams showing CVN2L0-B and CVN2L0-N binding to gp140 at the same analyte concentrations. (**B**) and (**C**) K_Ds for CVN and mutants (Table 1) are calculated according to the 1:1 binding model showing exact fits for both, the association phase = 190 sec; and the dissociation phase = 120 sec. (**D**) Sensorgrams for CVN–E binding to gp120 and gp140 (green). (**E**) Sensorgrams for CVN2L0-P binding to gp120 and gp140 (red). Analysis was as in B.

Surface Plasmon Resonance (SPR)

The SPR binding assays compared binding affinities of CV-N (2 disulfide bonds) and engineered dimeric binding site mutants (4 disulfide bonds) to gp120, gp140, HA, and Ebola GP1,2 using BIAcore T100 (see Materials and Methods).

Disulfide bond variant CVN2L0-V2 (3 disulfide bonds) was tested for binding affinities to HA by SPR on Reichert's SR7500DC (Buffalo, NY, USA, Figure S2B). Kinetic studies were performed on the two-channels SPR instrument using various analyte concentrations in the range of 10^{-5}–10^{-8} M, with a regeneration step after each injection and blank measurements after different analytes. CMD500D SPR sensorchips were purchased from Xantec bioanalytics (Düsseldorf, Germany). Running buffer (HBS-EP (+)) contained 10 mM HEPES, 150 mM NaCl, 3 mM EDTA and 0.05% Tween at pH 7.4. Measurements were performed at 25 °C at a flow rate of 30 µL/min. All solutions were degassed and filtered (0.2 µm) before injection into the system. Sensorchips were activated using single-channel amine coupling, where 0.4 M EDC/HCl and 0.1 M NHS were used to activate the carboxymethyl dextran hydrogel chip surface. The ligand (HA) was diluted to 20 µg/mL in 10 mM sodium acetate buffer (pH 5) and then injected onto the activated chip surface. The recombinant influenza A virus hemagglutinin H3 protein (H3N2 A/Wisconsin/67/05) was obtained from abcam (Cambridge, UK). The second channel, not coated with the ligand, served as a reference channel.

A)

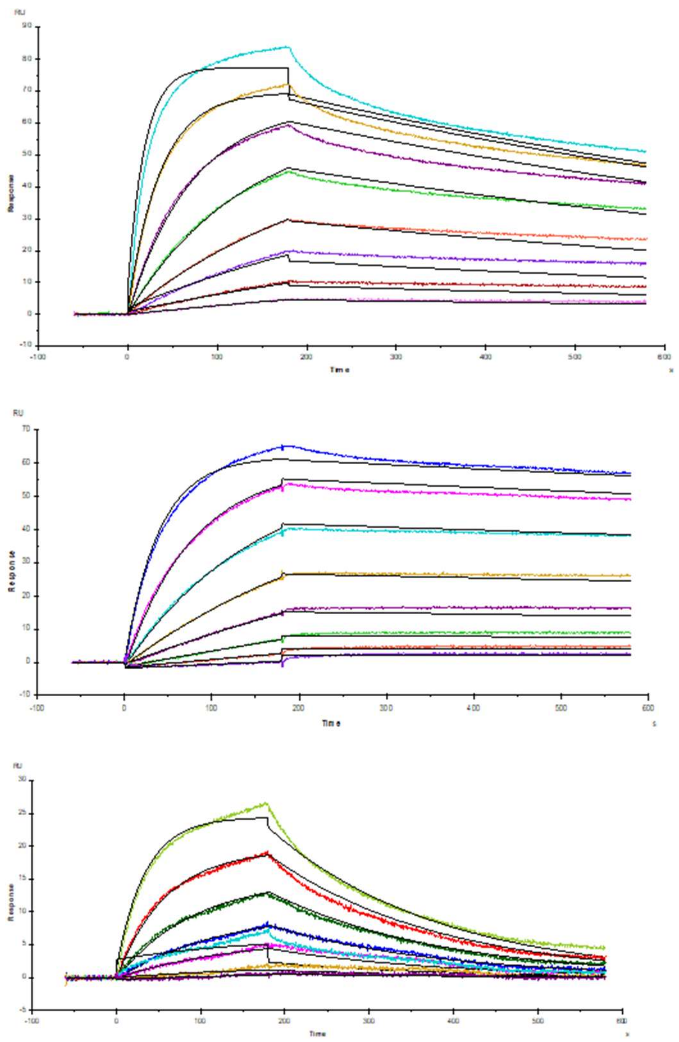

CV-N

K_D: 5.7 × 10^{-9} M

CVN2L0-B

K_D: 2.7 × 10^{-9} M

CVN2L0-N

K_D: 6.5 × 10^{-8} M

B)

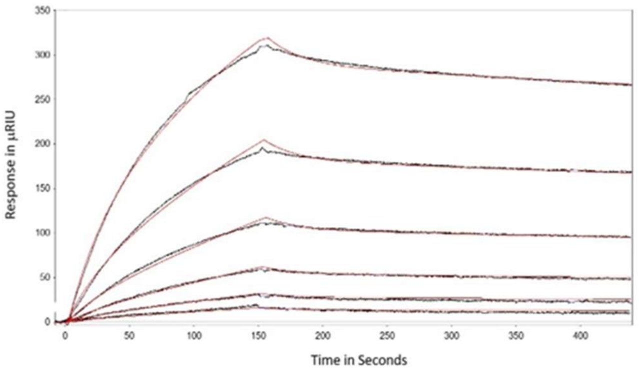

CVN2L0-V2

K_{D1}=49 nM and
K_{D2}=8 µM

Figure S2. Sensorgrams from conventional SPR experiments on BIAcore T100 and Reichert SR7500DC instrument. (**A**) CV-N, CVN2L0-B and CVN2L0-N binding to HA full length protein (A/New-York/55/04). Concentrations of analytes ranged from 5120 nM (2560, 1280, 640, 320, 160, 80, 40, 20, 10, 5) to 2.5 nM. (**B**) CVN2L0-V2 interactions with H3N2 A/Wisconsin/67/05 involving 1 high-affinity and 2 low-affinity binding sites (Schilling, P.E. et al., 2020). Concentrations of the CV-N variant ranged from 1 µM to 31.2 nM. The data were fit to a conformational change model in ClampTM with a K_D1 of 49 nM and a K_D2 of 8 µM.

A)

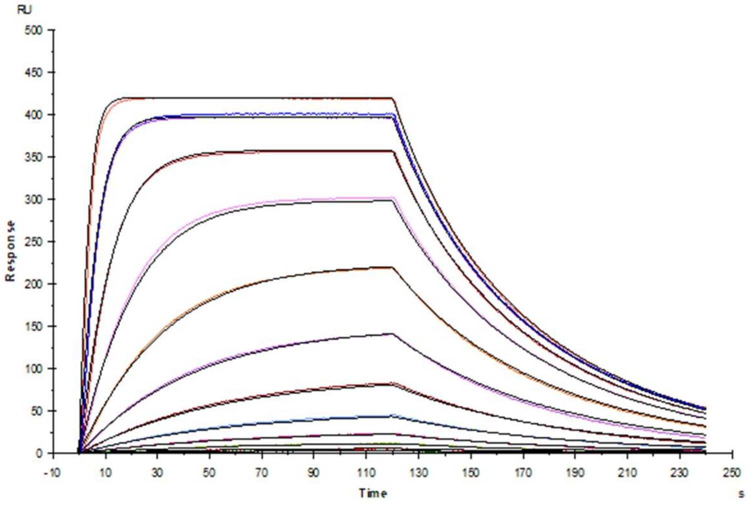

$K_D = 6.4 \times 10^{-8}$ M; $R_{max\ (calculated)} \approx 447.1$ RU

B)

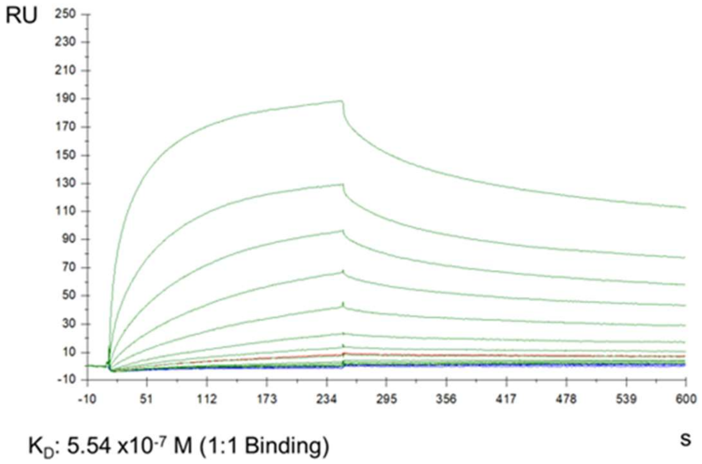

Figure S3. Surface Plasmon Resonance (**A**) Sensorgram from the binding analysis of HA H3 top (Fleury, D. et al., 1998) to HC19 Fab, which was captured by protein A on a pre-immobilized sensor S chip. (**B**) CV-N interaction with HA H3 top was verified on BIAcore T100.

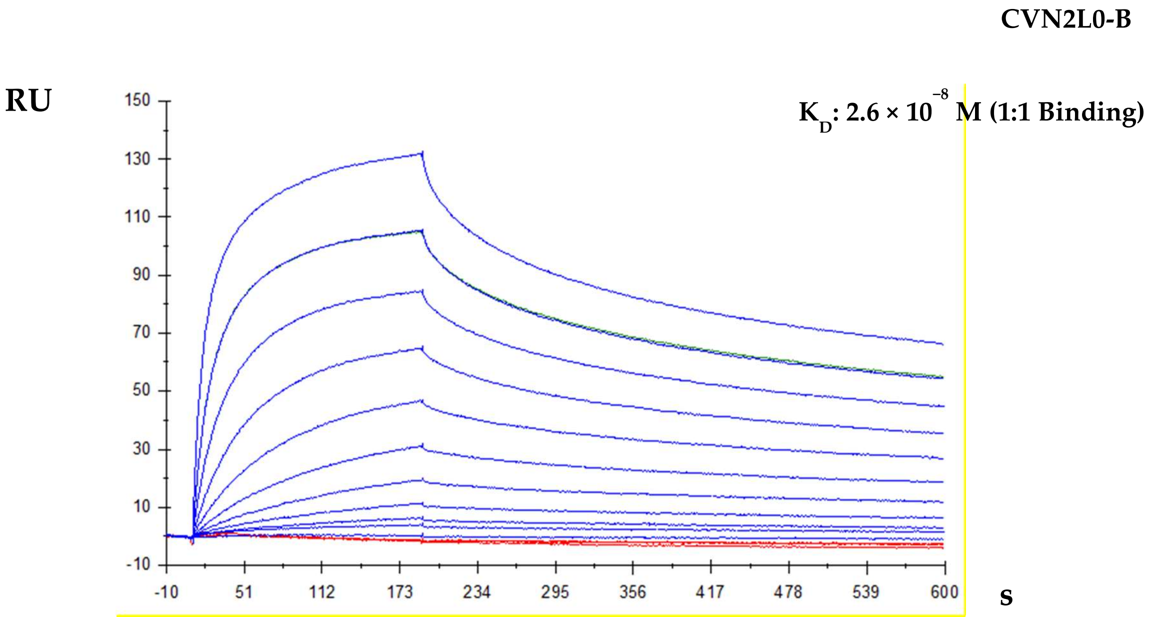

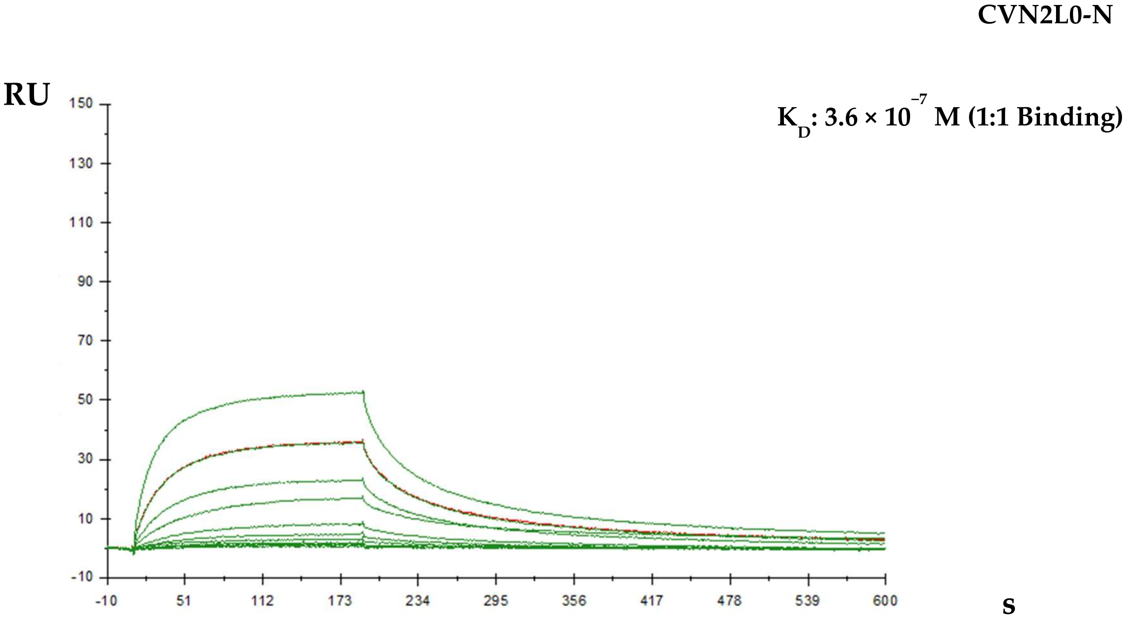

Figure S4. CVN2L0-B and CVN2L0-N binding curves are shown for Ebola GP1,2 which was immobilized onto CM5 sensorchips. Concentrations of analytes were 1280, 640, 320, 160, 80, 40, 20, 10, 5 and 2.5 nM (see above).

CVN2—No. of functional binding sites.

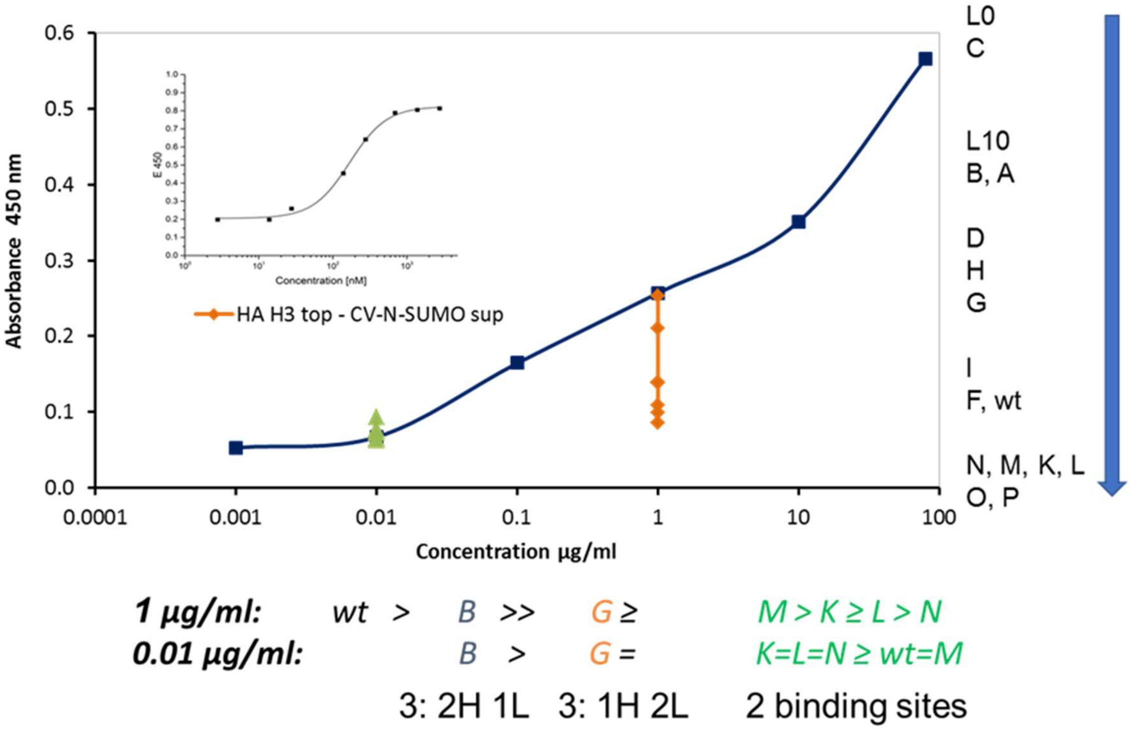

Figure S5. Calibration curve for ELISA testing CV–N affinities.

Binding to HA top was determined to be $K_D = 4.17 \times 10^{-8}$ M (CVN2L0), $K_D = 5.54 \times 10^{-7}$ M (WT CV–N), and $K_D = 1.25 \times 10^{-8}$ M (HC19 Fab).

Enzyme-Linked Immunosorbent Assay (ELISA)

The binding of engineered dimer CVN2L0 to hemagglutinin HA H3 was verified by applying ELISA in microtitration plate format. Briefly, CVN2L0 (without a linker between the two tandem-repeats of co-expressed CV–N monomers) was subcloned into pE-SUMO expression vector (T7, Amp) for *E.coli* (Life Sensors, Inc., US), including a His-tag for protein purification from the insoluble fraction, or supernatant. Additives were 10 mM $MgCl_2$, 1 mM $MgSO_4$ and glucose. The sequence encoding the same protein was cloned into pET27b(+) and re-analyzed by LC-ESI-MS/MS. Nunc Maxisorp microtitration plates were coated with HA H3 100–0.1 µg/mL, blocked with 1% bovine serum albumin, washed 3 times with PBS-Tween, and subsequently incubated with SUMO-CVN2L0 fusion protein. Anti-SUMO primary antibody (1:1,000) and secondary anti-Chicken IgY antibody conjugated with horseradish peroxidase (1:5,000) were used for read out. Washing steps (3–5 times) were applied after each incubation step.

Endpoint assay

The CV-N dimer with a linker of 10 residues between 2 repeats forming the domain-swapped CVN2 molecules (CVN2L10 with 2 high-affinity and 2 low-affinity carbohydrate binding sites) and binding site knockout mutants were created as described by Keeffe, J.R. et al. (2011), and were titered against the HA full-length protein at 1 and 0.01 µg/mL. The mutants were the following: CVN2L0, -L10, CVN2L0-A, C, D, F, H, I, which had 4 functional carbohydrate binding sites; 2 high-affinity (H) sites and 2 low-affinity (L) sites. CVN2L0-B and G had 3 functional binding sites. WT CVN and CVN2L0-M, N, L, K had 2, and CVN2L0-O, P had 1.

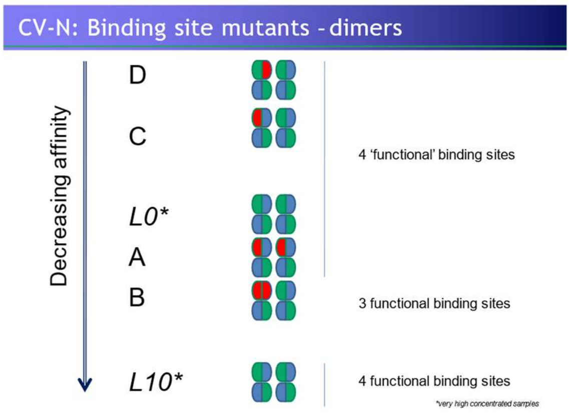

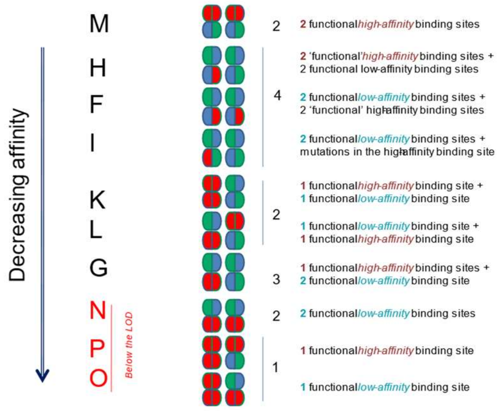

Half of the domains filled in red mark a mutated domain that resulted in binding-site knockouts and mutations in both domains of a single binding site in one or the other monomer.

Mutations	1st tandem-repeat CV-N sequence	2nd tandem-repeat CV-N sequence
CVN2L0$_{\Delta A}$ (-B)	K3N, T7A, E23I	N93A
CVN2L0ΔBB (-N)	E41A, N42A, T57A, R76A, Q78G	E41A, N42A, T57A, R76A, Q78G
CVN2L0ΔAAB (-P)	K3N, T7A, E23I, E41A, N42A, N93A	K3N, T7A, E23I, T57A, R76A, Q79G, N93A
CVN$_{\Delta A}$ (-E)	K3N, T7A, E23I, N93A	

References

Schilling, P. E., Kontaxis, G., Dragosits, M., Schiestl, R. H., Becker, C. F. W., and Maier, I. (2020) Mannosylated hemagglutinin peptides bind cyanovirin-N independent of disulfide-bonds in complementary binding sites. *RSC Adv* **10**, 11079–11087.

Fleury, D., Wharton, S. A., Skehel, J. J., Knossow, M., and Bizebard, T. (1998) Antigen distortion allows influenza virus to escape neutralization. *Nat Struct Biol* **5**, 119–123.

Keeffe, J. R., Gnanapragasam, P. N., Gillespie, S. K., Yong, J., Bjorkman, P. J., and Mayo, S. L. (2011) Designed oligomers of cyanovirin-N show enhanced HIV neutralization. *Proc Natl Acad Sci USA* **108**, 14079–14084.
